# Reverse-migrated neutrophils regulated by JAM-C are involved in acute pancreatitis-associated lung injury

**DOI:** 10.1038/srep20545

**Published:** 2016-02-04

**Authors:** Deqing Wu, Yue Zeng, Yuting Fan, Jianghong Wu, Tunike Mulatibieke, Jianbo Ni, Ge Yu, Rong Wan, Xingpeng Wang, Guoyong Hu

**Affiliations:** 1 Department of Gastroenterology, Shanghai Tenth People’s Hospital, Tongji University School of Medicine, Shanghai, China; 2 Department of Gastroenterology, Shanghai General Hospital, Shanghai Jiaotong University School of Medicine, Shanghai, China

## Abstract

Junctional adhesion molecule-C (JAM-C) plays a key role in the promotion of the reverse transendothelial migration (rTEM) of neutrophils, which contributes to the dissemination of systemic inflammation and to secondary organ damage. During acute pancreatitis (AP), systemic inflammatory responses lead to distant organ damage and typically result in acute lung injury (ALI). Here, we investigated the role of rTEM neutrophils in AP-associated ALI and the molecular mechanisms by which JAM-C regulates neutrophil rTEM in this disorder. In this study, rTEM neutrophils were identified in the peripheral blood both in murine model of AP and human patients with AP, which elevated with increased severity of lung injury. Pancreatic JAM-C was downregulated during murine experimental pancreatitis, whose expression levels were inversely correlated with both increased neutrophil rTEM and severity of lung injury. Knockout of JAM-C resulted in more severe lung injury and systemic inflammation. Significantly greater numbers of rTEM neutrophils were present both in the circulation and pulmonary vascular washout in JAM-C knockout mice with AP. This study demonstrates that during AP, neutrophils that are recruited to the pancreas may migrate back into the circulation and then contribute to ALI. JAM-C downregulation may contribute to AP-associated ALI via promoting neutrophil rTEM.

Acute pancreatitis (AP) is an acute inflammatory disease of the pancreas that, in its severe form, affects nearly all body systems^1^. In severe acute pancreatitis (SAP), a systemic inflammatory response leads to distant organ damage and the development of multiple organ dysfunction syndromes (MODS). According to the revised Atlanta classification system, SAP is characterized by persistent organ failure. Patients who develop persistent organ failure within the first few days of the disease are at an increased risk of death, and the mortality has been reported to be as high as 36–50%^2^,^3^,^4^. Among these cases, acute lung injury (ALI) appears to be the predominant cause of death^5^.

The mechanisms underlying ALI are still poorly understood. Evidence has been accumulating that cytokines and chemokines may play major roles in the pathogenesis of respiratory complications following AP^6^. Intravenous administration of pancreatic ascites in healthy rats has been shown to induce lung injury^7^, while ascites fluid collected from rats with AP failed to induce lung injury in IL-1β/TNF-α receptor double knockout mice^8^. Cytokine neutralisation also attenuates the systemic stress response and is associated with a modest decrease in mortality in AP^9^. In addition, macrophages, and in particular peritoneal macrophages, alveolar macrophages and Kupffer cells, can also be activated during AP. Activated macrophages release systemic cytokines and inflammatory mediators that contribute to the systemic inflammatory response and to lung injury associated with AP^10^,^11^,^12^,^13^,^14^,^15^. Administration of CNI-1493, a compound that inhibits macrophage activation, prior to the induction of SAP in rats results in increased survival and reduced disease progression^16^,^17^. Another population of leukocytes that are involved in AP-associated ALI is neutrophils. Neutrophils are the first cells of the immune system to be recruited to a site of injury or inflammation and are thought to play a key role in the progression of ALI^18^,^19^,^20^,^21^. Lung injury results from local pulmonary endothelial cell injury secondary to neutrophil-generated oxygen-radical products^22^. The depletion of neutrophils prevents increases in microvascular permeability and myeloperoxidase (MPO) levels in the lungs in experimental pancreatitis^22^,^23^.

Although neutrophils are widely considered to play an important role in the development of ALI associated with AP, the molecular mechanisms of their activities remain unclear. Neutrophils are able to migrate through endothelial cell (EC) junctions in an abluminal-to-luminal direction, in a process known as reverse transendothelial migration (rTEM). Previous studies have shown that neutrophils that have reverse migrated have a greater ability to generate reactive oxygen species (ROS), which contribute to local tissue damage^24^. Recently, Woodfin and colleagues reported that junctional adhesion molecule-C (JAM-C) plays a key role in supporting neutrophil rTEM *in vivo*^25^. The presence of these cells contributes to the dissemination of systemic inflammation and is associated with secondary organ and tissue damage such as lung injury^25^. However, no study published to date has examined the role of neutrophil rTEM in AP-associated lung injury.

In this study, we investigate whether the neutrophil rTEM is involved in AP-associated ALI and determine the role of JAM-C in this process. We observed elevated rTEM neutrophils in AP-associated ALI both in murine experimental AP and in human patients. Pancreatic JAM-C expression was decreased in mice with experimental AP and lung injury. JAM-C knockout mice exhibit severe lung injury and systemic inflammation in experimental AP. A significantly higher proportion of rTEM neutrophils can be found both in the peripheral blood and in pulmonary vascular washout fluid from JAM-C knockout mice compared with wild-type animals. These results suggest that JAM-C contributes to ALI via the regulation of neutrophil rTEM in AP.

## Results

### Neutrophil rTEM positively correlated with AP-associated lung injury

**Figure 1:**
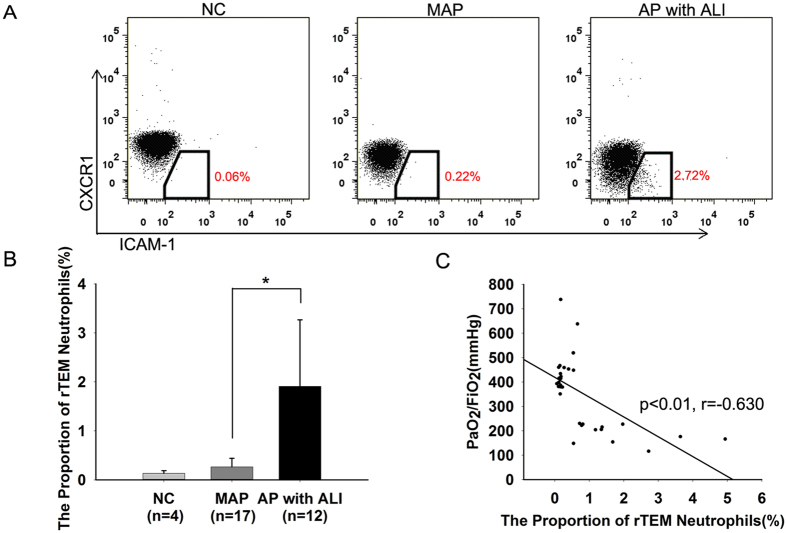
Reverse-migrated (rTEM) neutrophils are positively associated with lung injury in patients with acute pancreatitis (A) Human neutrophils were analyzed for ICAM-1 and CXCR1 expression by flow cytometry; examples of scatter plots of ICAM-1 and CXCR1 profiles are shown. The numbers in red demonstrate the greater percentage of ICAM-1^high^CXCR1^low^ population (rTEM neutrophils) in patients with acute pancreatitis (AP) associated acute lung injury (ALI). (**B**) A comparison of the levels of rTEM neutrophils in healthy controls (NC, n = 4), MAP (mild acute pancreatitis, n = 17) and AP with ALI (n = 12) (*p < 0.01). (**C**) An increased level of rTEM neutrophils in the peripheral blood positively correlated with the decline of PaO_2_/FiO_2_ (r = −0.630, *p* < 0.01).

Neutrophils that have undergone rTEM have a distinctive ICAM-1^high^CXCR1^low^ phenotype in contrast to neutrophils of the blood and bone marrow and those that have undergone normal transmigration, all of which are ICAM-1^low^ 24,25. First, we tested whether this subtype of neutrophils is involved in lung injury in human AP. As expected, rTEM neutrophils were significantly more prevalent in patients with ALI compared with healthy volunteers and those with mild acute pancreatitis (MAP) (Fig. 1A,B). The increased proportion of rTEM neutrophils found in the circulation was related to a decrease in PaO_2_/FiO_2_ (Fig. 1C).

**Figure 2:**
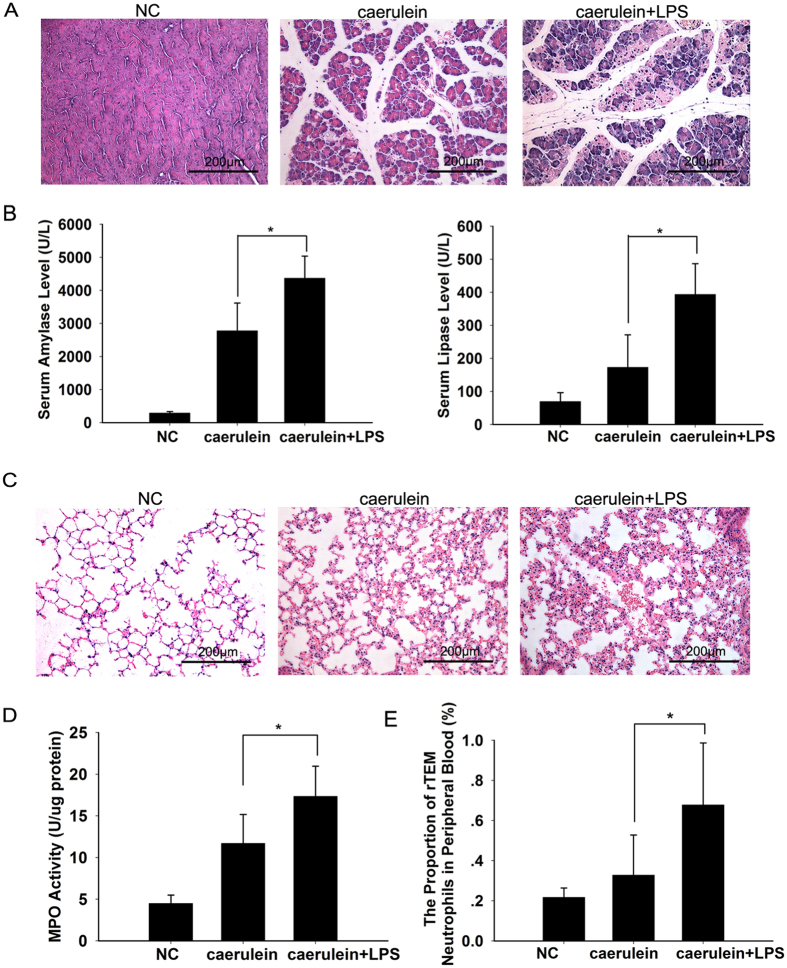
Pancreatic inflammation and lung injury were more apparent in caerulein and LPS-induced pancreatitis. (**A**) Representative micrographs of pancreatic tissue following caerulein and LPS-induced pancreatitis and single caerulein-induced pancreatitis are presented. (**B**) Serum concentration of amylase and lipase show pancreatic injury was more apparent in caerulein and LPS-induced pancreatitis (*p < 0.01). (**C**) Representative micrographs of lung tissue following caerulein and LPS-induced pancreatitis and single caerulein-induced pancreatitis are presented. (**D**) Myeloperoxidase (MPO) levels from lung tissue show lung injury was more apparent in caerulein and LPS-induced pancreatitis (*p < 0.05). (**E**) Significantly higher proportion of rTEM neutrophils in murine peripheral blood was observed in caerulein and LPS-induced pancreatitis (*p < 0.05). NC: normal control, n = 6 for each group. Original magnification ×200. Mice were sacrificed 12 h after the first caerulein injection.

**Figure 3:**
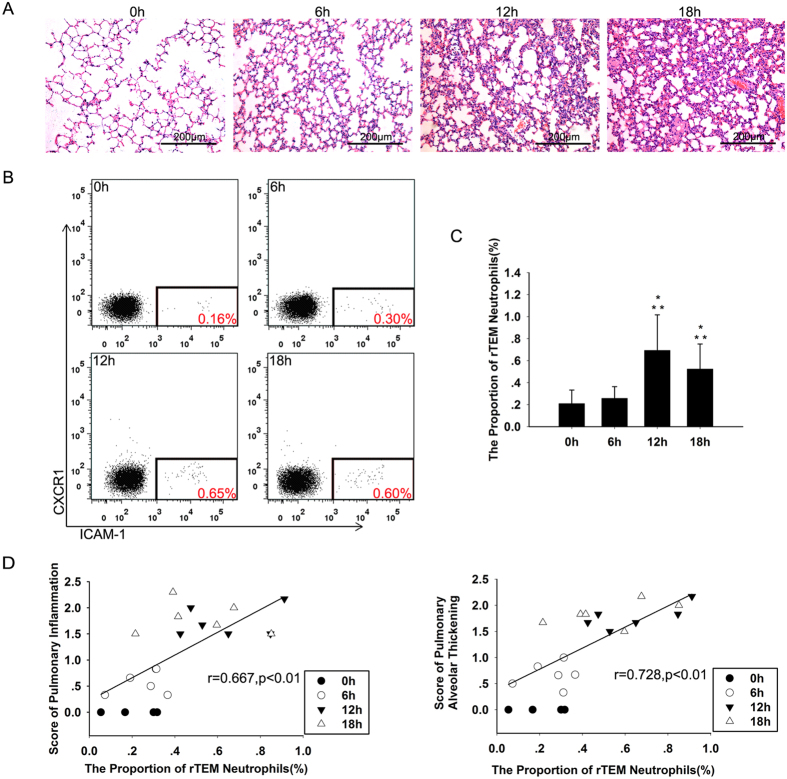
Reverse-migrated (rTEM) neutrophils are associated with lung injury in a murine model of experimental pancreatitis induced by caerulein (100 μg/kg) in combination with LPS (5 mg/kg). (**A**) Representative micrographs of lung tissue from control mice and mice with AP are presented. Lung tissues were taken 0h, 6 h, 12 h and 18 h after the first caerulein injection. (**B**) Murine neutrophils were analyzed for ICAM-1 and CXCR1 expression by flow cytometry. Example scatter plots of ICAM-1 and CXCR1 profiles are shown. The numbers in the red field show the proportion of the rTEM neutrophils, which increased significantly at 12 h and 18 h. (**C**) A comparison of the proportion of rTEM neutrophils in different time points (*p < 0.05 compared to the 0h group, **p < 0.05 compared to the 6h group, n = 6 at each time point). (**D**) An increased level of rTEM neutrophils in the peripheral blood positively correlated with score of lung injury (n = 6 at each time point; alveolar thickening: r = 0.728, *p* < 0.01; inflammation: r = 0.667, *p* < 0.01). Original magnification ×200. Mice were sacrificed 0 h, 6 h, 12 h and 18 h after the first caerulein injection.

Furthermore, we established a murine experimental model of AP. To obtain a more severe model of AP, mice were given a high dose (100 μg/kg) of caerulein and one intraperitoneal injection of lipopolysaccharide (LPS, 5 mg/kg) immediately after the final injection of caerulein. In this setting, pancreatic inflammation and lung injury associated with AP became more severe than single use of caerulein (100 μg/kg) (Fig. 2A–D, Table 1). Definite ALI was observed at both 12 h and 18 h after the administration of caerulein (Fig. 3A, Table 2). The proportion of neutrophils that had undergone rTEM in murine peripheral blood was also significantly elevated at the same time points (Fig. 3B,C). Higher proportion of rTEM neutrophils was also observed when compared with the group of single use of caerulein (Fig. 2E). Histological scores showed that observed lung injuries became increasingly severe as the proportion of rTEM neutrophils increased (Fig. 3D).

Together, these results indicate that elevated proportion of rTEM neutrophil in peripheral blood were positively correlated with the severity of lung injuries in both human and murine AP.

### Expression of JAM-C is downregulated in caerulein and LPS-induced pancreatitis

**Figure 4:**
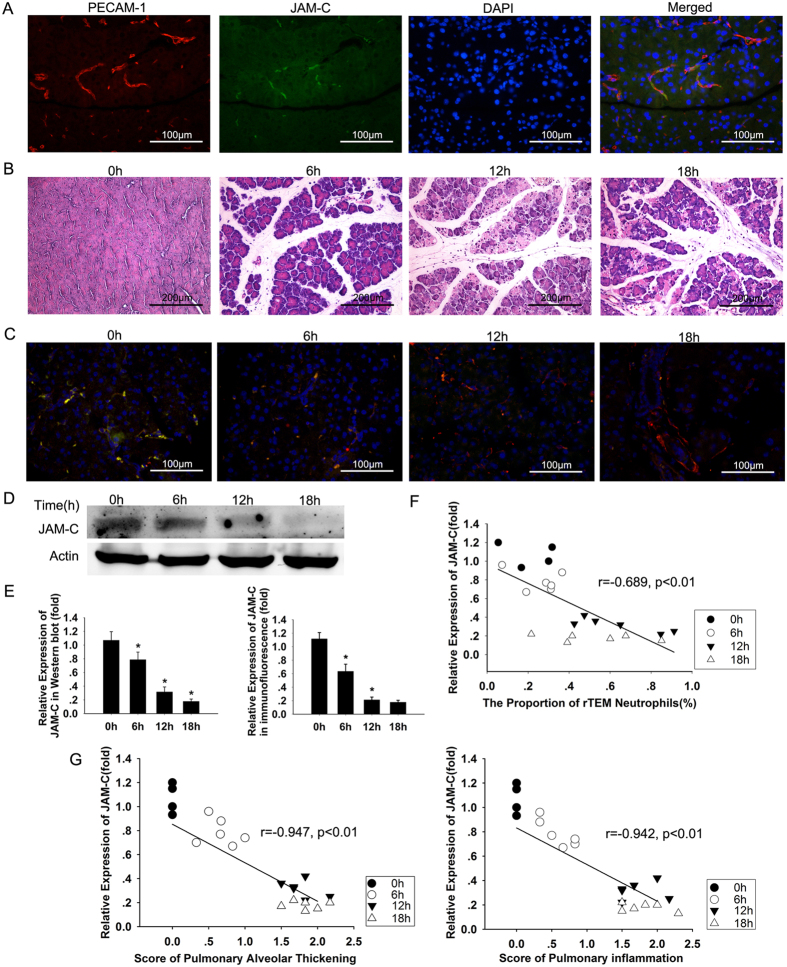
Expression of JAM-C in pancreas and its relationship between reverse-migrated neutrophils and lung injury during caerulein and LPS-induced pancreatitis. (**A**) Double immunofluorescent staining for PECAM-1 (red) and JAM-C (green) in normal murine pancreas. DAPI (blue) was used to counterstain the nuclei. JAM-C is expressed in blood vessels in the murine pancreas. (**B**) Representative micrographs of the pancreas of control mice and mice with experimental pancreatitis at 0 h, 6 h, 12 h and 18 h after the first caerulein injection are shown. (**C**,**D**) The level of JAM-C expression is downregulated at both 12 h and 18 h after the first caerulein injection. JAM-C protein expression (green) with a co-staining for PECAM-1 (red) is shown on immunofluorescence (**C**) and a western blot of total pancreatic lysates (**D**). (**E**) A quantitative analysis of the western blot (left) and immunofluorescence (right) showed JAM-C was down-regulated in a time dependent manner (*p < 0.01 compared with the previous time point). (**F**) A decreased level of JAM-C correlated with increased numbers of rTEM neutrophils in the peripheral blood (r = −0.689, *p* < 0.01). (**G**) A decreased level of JAM-C correlated with lung injury (alveolar thickening: r = −0.947, *p* < 0.01; inflammation: r = −0.942, *p* < 0.01). n = 6 at each time point. Original magnification: ×400 for immunofluorescence, ×200 for H&E sections. Mice were sacrificed 0 h, 6 h, 12 h and 18 h after the first caerulein injection.

Previous study showed JAM-C was downregulated and increased neutrophil rTEM in ischemia-reperfusion injury^25^. To determine the role of JAM-C in neutrophil rTEM during experimental pancreatitis, we investigated the expression of JAM-C in the pancreas in caerulein and LPS-induced pancreatitis. Double staining for the endothelial marker PECAM-1 and JAM-C showed that JAM-C is expressed in pancreatic blood vessels (Fig. 4A), consistent with the results of a previous study^26^. After the induction of AP, remarkable interstitial oedema and necrosis of acinar cells were observed, together with significant inflammatory cell infiltration at 12 h and 18 h after the induction of AP (Fig. 4B). JAM-C expression levels decreased at both 12 h and 18 h after the induction of AP compared with control animals (Fig. 4C–E, Supplementary Figure S1). These findings suggest that JAM-C expression in the pancreas is downregulated in caerulein and LPS-induced pancreatitis.

### Decreased JAM-C expression is associated with lung injury and increased neutrophil rTEM

After characterizing the changes in reverse-migrated neutrophils and the expression of JAM-C in AP-associated ALI, we investigated the relationship between the expression level of JAM-C and rTEM neutrophil. As shown in Fig. 4F, JAM-C downregulation was associated with elevated peripheral rTEM neutrophil. Reduced levels of JAM-C expression were also associated with increased histological scores for lung injury (Fig. 4G). As an increased level of rTEM neutrophil correlated with the severity of lung injury, these results suggest that during SAP, a decreased level of JAM-C expression may result in a high level of rTEM neutrophils in circulation, which contributes to ALI.

### JAM-C deficiency does not affect pancreatic injury in murine experimental acute pancreatitis

**Figure 5:**
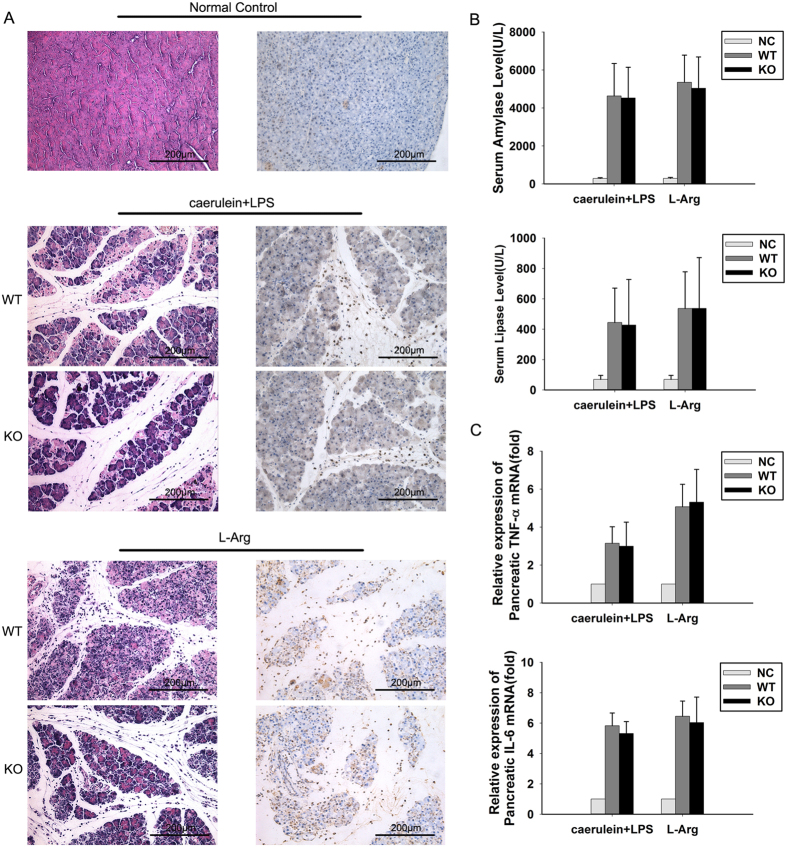
JAM-C knockout does not alleviate murine experimental pancreatitis. (**A**) Representative micrographs show no significant differences in pancreatic injuries between JAM-C-deficient mice and wild-type mice in caerulein plus LPS or L-Arg induced pancreatitis. Left row: H & E sections; Right row: immunohistochemical analysis of neutrophil infiltration by Ly-6G antibody. No significant differences were observed in the activities of serum amylase or lipase (**B**) or in the mRNA levels of IL-6 and TNF-α in pancreatic tissues (**C**). NC: normal control, WT: wild-type mice, KO: JAM-C knockout mice. n = 4~11. Original magnification ×200. Mice were sacrificed 12 h after the first caerulein injection or 3 d after the first L-arginine (L-Arg) injection.

To further explore the role of JAM-C in the regulation of neutrophil rTEM, we established experimental AP in JAM-C-deficient (JAM-C^−/−^) mice. Firstly, murine AP was induced by caerulein and LPS. Unexpectedly, no significant difference was found in the extent of pancreatic damage or in the degree of neutrophil infiltration between JAM-C^−/−^ mice and wild-type mice (Fig. 5A, Table 3). No significant difference in serum levels of amylase and lipase were observed between JAM-C^−/−^ and wild-type mice (Fig. 5B).

Leukocytes in inflammatory tissue become activated and produce a series of inflammatory cytokines that contribute to local tissue damage. Thus, we performed qRT-PCR on pancreatic tissue samples to evaluate the levels of TNF-α and IL-6 mRNA. In agreement with the histological findings, no significant difference in the levels of inflammatory cytokines was observed between JAM-C^−/−^ and wild-type mice (Fig. 5C).

In order to exclude specific differences between models, we also established L-arginine (L-Arg)-induced AP. Again, unchanged histological scores, serum concentrations of amylase and lipase and levels of TNF-α and IL-6 mRNA were found between JAM-C^−/−^ and wild-type mice (Fig. 5).

### JAM-C deficiency aggravates lung injuries and systemic inflammation in experimental pancreatitis

**Figure 6:**
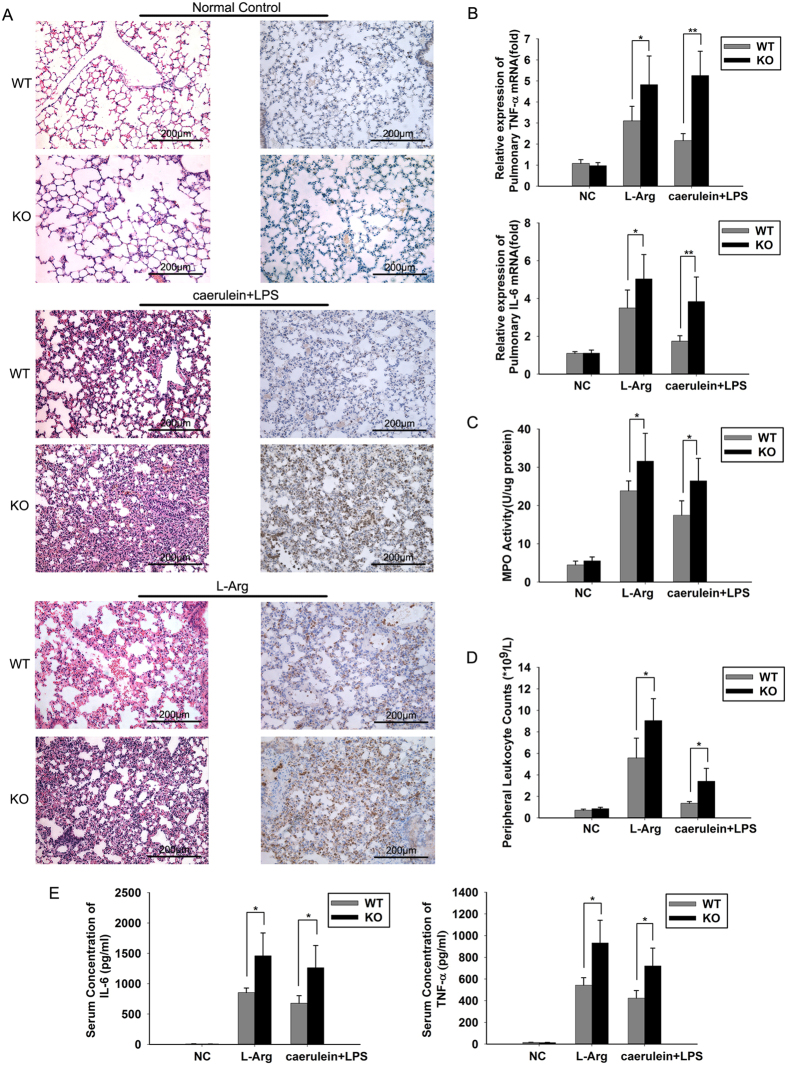
JAM-C deficiency aggravated lung injuries and systemic inflammation in experimental pancreatitis. (**A**) Representative micrographs show that JAM-C-deficient mice experienced more severe lung injuries than their wild-type littermates. Left row: H&E sections; Right row: immunohistochemical analysis of neutrophil infiltration by Ly-6G antibody. (**B**) Quantitative reverse transcription PCR demonstrated increased mRNA levels of IL-6 and TNF-α in the lung tissues of JAM-C-deficient mice (*p < 0.05, **p < 0.01). (**C**) Pulmonary myeloperoxidase (MPO) activity showed that JAM-C-deficient mice exhibited more severe lung damage than wild-type animals (*p < 0.05). (**D**) Total counts of circulating leukocytes showed increased numbers of leukocytes in JAM-C-deficient mice compared with their wild-type littermates in murine acute pancreatitis (*p < 0.01). (**E**) The serum levels of IL-6 and TNF-a were increased in JAM-C-deficient mice as measured by ELISA (*p < 0.05). NC: normal control, WT: wild-type mice, KO: JAM-C knockout mice. n = 4~11. Original magnification ×200. Mice were sacrificed 12 h after the first caerulein injection or 3 d after the first L-arginine (L-Arg) injection.

Interestingly, we found that lung injuries were more severe in the JAM-C^−/−^ mice than in the wild-type mice following two models of AP. The severity of lung damage and the extent of leukocyte infiltration were significantly increased in JAM-C^−/−^ mice (Fig. 6A, Table 4). The levels of inflammatory cytokines and MPO activity in the lungs were also significantly higher in JAM-C^−/−^ mice (Fig. 6B,C). Because local inflammation is accompanied by transient leukocytosis in the blood, we then examined the numbers of circulating leukocytes after AP induction. The results showed the numbers of circulating leukocytes were significantly increased in JAM-C^−/−^ mice (Fig. 6D). In addition, the serum levels of TNF-α and IL-6 were also significantly higher in JAM-C^−/−^ mice (Fig. 6E), indicating that systemic inflammation is more severe after the loss of JAM-C.

### An increase in neutrophil rTEM is enhanced in JAM-C-deficient mice

**Figure 7:**
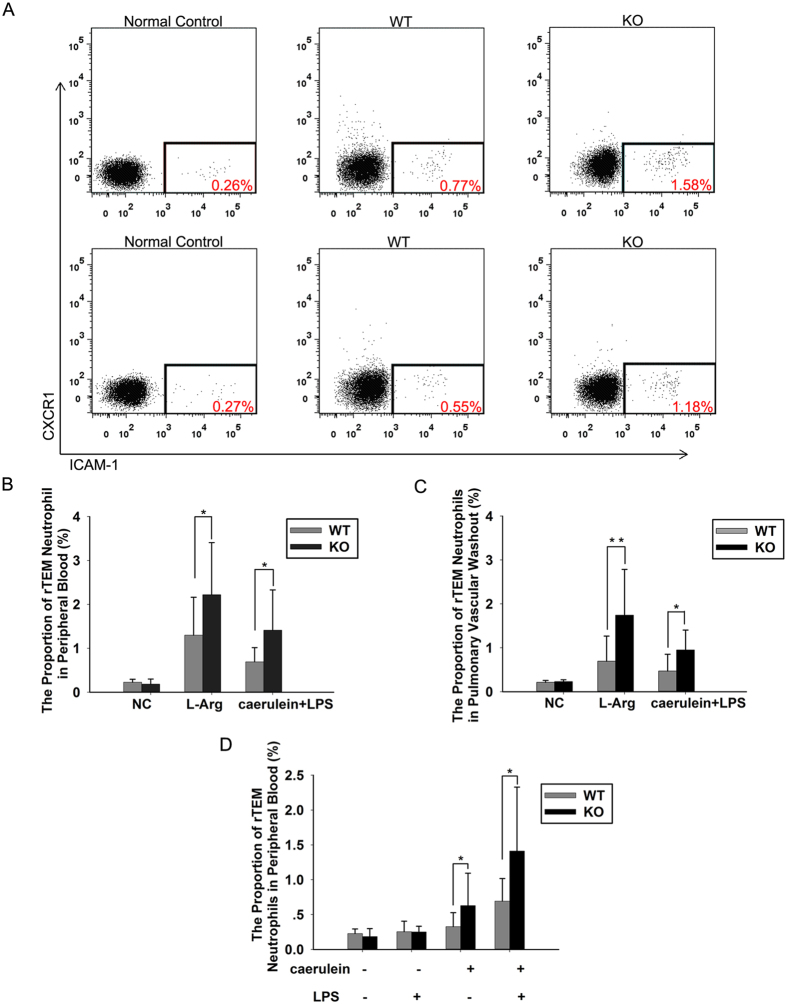
The number of reverse-migrated neutrophils was increased in JAM-C-deficient mice. (**A**) Murine neutrophils were analyzed for ICAM-1 and CXCR1 expression in peripheral blood (above) and in the pulmonary vascular washout (below) by flow cytometry. Examples of scatter plots of ICAM-1 and CXCR1 profiles are shown. The numbers in red demonstrate the greater percentage of rTEM neutrophils in JAM-C-deficient mice. The proportion of rTEM neutrophils in peripheral blood (**B**) and pulmonary vascular washouts (**C**) in JAM-C-deficient mice was significantly higher than in wild-type mice (*p < 0.05, **p < 0.01). (**D**) Single use of LPS was not able to induce high percentage of rTEM neutrophils in peripheral blood (*p < 0.05). NC: normal control, WT: wild-type mice, KO: JAM-C knockout mice. n = 4~11. Mice were sacrificed 12 h after the first caerulein injection or 3 d after the first L-arginine (L-Arg) injection.

We have found that JAM-C downregulation may result in increased neutrophil rTEM in peripheral blood during AP. In agreement with this finding, significantly higher proportion of reverse-migrated neutrophils in peripheral blood was observed in JAM-C-deficient mice than in wild-type mice in response to AP (Fig. 7A,B). Similar results were obtained in neutrophils from pulmonary vascular washouts (Fig. 7A,C).

**Figure 8:**
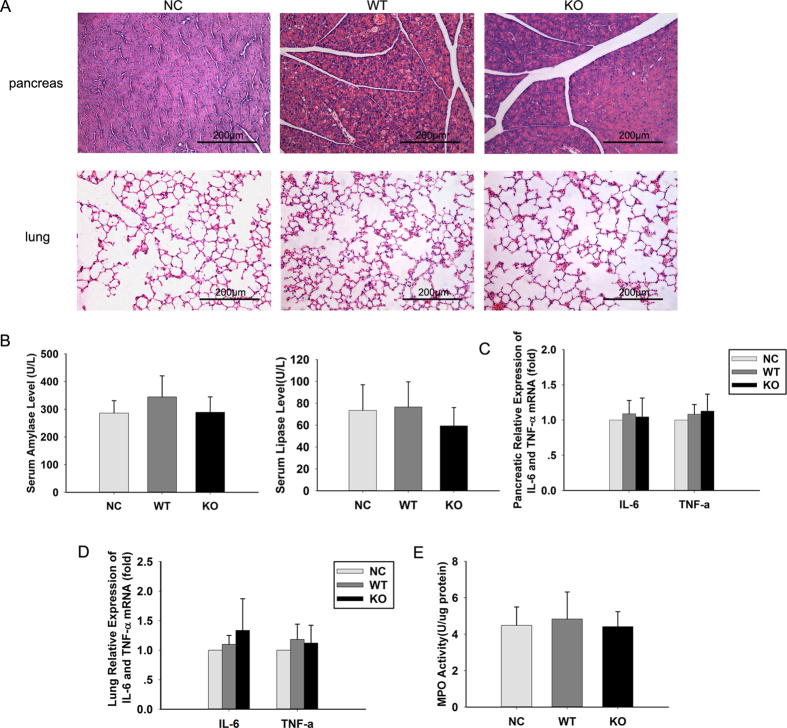
Single use of LPS was not able to induce pancreatic and lung damage in mice. (**A**) Representative micrographs of pancreas and lung showed that single use of LPS was not able to induce pancreatic and lung damage. Activities of serum amylase and lipase (**B**), the mRNA levels of IL-6 and TNF-α in pancreatic tissues (**C**) or lung tissues (**D**) and pulmonary MPO activity (**E**) indicate single use of LPS is not able to induce pancreatic or pulmonary injury in both wild-type and JAM-C knockout mice. NC: normal control (n = 4), WT: wild-type mice (n = 7), KO: JAM-C knockout mice (n = 7). Original magnification ×200. The mice were sacrificed 2 h after the LPS injection.

Previous study reported LPS stimulated the expression of ICAM-1 on neutrophils *in vitro*^27^,^28^. To explore in our study whether the very high proportion of ICAM-1^high^ neutrophils in the circulation is a result of activation by LPS, we observed the proportion of ICAM^high^CXCR1^low^ neutrophils by single use of LPS. In this experiment, mice were given ten hourly intraperitoneal injections of saline instead of caerulein first, LPS (5 mg/kg) was administered immediately after final injection of saline. Mice were sacrificed 12h after the first saline injection. The results showed that single use of LPS could only slightly increase the number of ICAM^high^CXCR1^low^ neutrophils which was far less than caerulein and LPS-induced pancreatitis or single use of caerulein in both wild-type and JAM-C^−/−^ mice. Besides, no difference was observed in the number of ICAM^high^CXCR1^low^ neutrophils between the two groups of mice (Fig. 7D). In addition, single use of LPS was not able to induce distinctive pancreatic or pulmonary damage in both wild-type and JAM-C^−/−^ mice (Fig. 8). These results indicated the majority of ICAM^high^CXCR1^low^ neutrophils from the blood may not due to the use of LPS.

Taken together, these results suggest that in the murine model of AP, JAM-C downregulation may promote an increase in the rTEM of neutrophils from the inflamed pancreas and these neutrophils re-enter the circulation and contribute to AP-associated ALI.

## Discussion

During the response to infection or injury, neutrophils are rapidly recruited from the circulation to sites of inflammation. However, the neutrophils in inflamed tissues can migrate in a retrograde direction across endothelial cells; these cells, moreover, constitute a population of tissue-experienced neutrophils with a distinct surface phenotype (ICAM-1^high^CXCR1^low^) and are referred as reverse-migrated neutrophils. It has been reported that neutrophils that have undergone reverse transendothelial migration (rTEM) are unlikely to be able to re-enter inflamed tissue. The apoptosis of rTEM neutrophils is delayed, which means that these cells are “long-lived” in the circulation^24^. More importantly, rTEM neutrophils have been demonstrated to have a greater ability to generate reactive oxygen species (ROS), which contribute to local tissue damage. The characteristics of rTEM neutrophils suggest the possibility that these cells can contribute to the systemic dissemination of inflammation in some types of inflammatory diseases. In acute pancreatitis (AP), acute lung injury (ALI) is the main factor contributing to early death^5^,^29^,^30^. Several studies have shown that ALI is the consequence of the systemic inflammatory response^31^,^32^. Although the role of neutrophils in AP-associated lung injury has been of significant interest, the exact molecular mechanism underlying the effects of these cells remains unclear. In the present study, we provided evidence for the first time that rTEM neutrophils may play an important role in the development of AP-associated lung injury.

Firstly, through an analysis of the peripheral blood from patients with AP, we found the levels of rTEM neutrophils were significantly increased in patients with ALI compared with both healthy volunteers and patients with mild acute pancreatitis (MAP). As the disorder progressed, the proportion of rTEM neutrophils in the circulation gradually increased, along with the severity of the lung injuries. These results suggest that during severe acute pancreatitis (SAP), neutrophils that are recruited to the pancreas may migrate back into the circulation, enter the pulmonary vessels and contribute to lung damage. Reverse-migrated neutrophils have also been reported in peripheral blood from patients with active RA and patients with severe atherosclerotic disease^24^. In the murine cremaster ischemia-reperfusion (I-R) model, a high percentage of rTEM neutrophils were found in the pulmonary vasculature, and this finding was associated with I-R-induced lung inflammation^25^. Together, these results suggest that reverse-migrated neutrophils are important in the pathology of some diseases and may play a critical role in the persistence of inflammation in humans, such as in AP-associated ALI.

However, how neutrophils undergo reverse migration is poorly understood. Recently, it was reported that JAM-C, a type of glycoprotein that is primarily expressed in vascular endothelial cells, regulates this process^25^. One study reported that in a murine cremaster I-R model, neutrophil rTEM occurred where JAM-C expression at EC junctions was reduced. When the interaction of JAM-C with JAM-B (another member of the JAM family which maintains JAM-C at endothelial cell junctions) is blocked, the number of neutrophils that reverse migrated increased. The same phenomenon has also been observed in endothelial cell-specific JAM-C-deficient mice^25^. In addition, another study showed that anti-JAM-C blocking mAbs induced the reverse transmigration of monocytes *in vitro* and *in vivo*^33^.

In order to make the pancreatitis more severe, we established the AP model with a higher dose of caerulein (100 μg/kg) plus LPS (5 mg/kg). In this setting, pancreatic inflammation and lung damage was apparent. JAM-C expression began to decrease 12 h after the induction of pancreatitis, which is associated with the extent of lung injury. However, Vonlaufen and colleagues reported that JAM-C is upregulated in single caerulein-induced (50 μg/kg) AP^26^. In our pilot study, single use of caerulein (50 μg/kg) was not able to induce apparent pancreatic inflammation (data not shown). We hypothesize that the extent of inflammation leads to different patterns of JAM-C expression. Further studies are needed to clarify the mechanism behind this phenomenon and by which JAM-C regulates neutrophil migration in cases of pancreatitis of different severity.

To further investigate the mechanisms of rTEM neutrophils in AP with ALI, we examined a JAM-C knockout mouse model. JAM-C is currently considered to be an adhesion molecule that controls the transmigration of leukocytes. Antibodies against JAM-C and soluble JAM-C both efficiently block the recruitment of leukocytes in several inflammatory models *in vivo*, including cutaneous inflammation, peritonitis and lung inflammation^34^,^35^,^36^. It was reported that blocking JAM-C with anti-JAM-C antibody blocks the pancreatic leukocyte infiltration of caerulein-induced AP in mice^26^. However, in our study, the deletion of JAM-C did not alleviate murine experimental pancreatitis. Similar results were reported in JAM-C-deficient mice with LPS-induced lung injury^37^. Aurrand-Lions *et al.* reported that the recruitment of granulocytes to the inflammatory site is only transiently affected by antibodies against JAM-C^35^, indicating different ways to deleting JAM-C may result in different patterns in the recruitment of leukocyte in inflammation. In addition to JAM-C, molecules such as PECAM-1, ICAM-1, VCAM and CD99 also contribute to the transendothelial migration of leukocytes from circulation to the inflammatory site during an inflammatory response. Thus, it is possible that the targeting of JAM-C with antibodies may act only on the early step of leukocyte recruitment before the upregulation of other endothelial adhesion molecules such as ICAM-1 or VCAM. Alternatively, antibodies that are directed against JAM-C may transduce signals inside endothelial cells which in turn affect the interactions between leukocytes and endothelial cells^38^,^39^. Moreover, a compensatory increase in the levels of those other molecules involved in the recruitment of leukocytes could exist in JAM-C-deficient mice. Further research would be necessary to address this question.

Importantly, we found that JAM-C deficiency leads to more severe lung injury when AP is induced. A significantly higher percentage of rTEM neutrophils were detected in both peripheral blood and pulmonary vascular washout in JAM-C-deficient mice compared with wild-type mice. Similar results were also observed in L-arginine induced-AP. These results provide evidence that rTEM neutrophils play an important role in AP-associated ALI. Neutrophils that are recruited to inflamed pancreatic tissue may undergo reverse transmigration when JAM-C expression falls, as occurs in SAP.

LPS was able to induce the expression of ICAM-1 on neutrophils *in vitro*^27^,^28^. However, we found that single use of LPS by intraperitoneal injection can only slightly increase the number of rTEM neutrophils, which was far less than caerulein and LPS or arginine-induced pancreatitis in both wild-type and JAM-C-deficient mice. So the majority of rTEM neutrophils from the blood in murine AP models may not due to the use of LPS. In addition, lung or pancreas injury was not observed in single use of LPS, which suggest pulmonary damage resulted from pancreatitis itself.

Moreover, we found that circulating leukocyte counts were increased in JAM-C-deficient mice, which may in part be due to the elevated numbers of reverse-migrated neutrophils in the peripheral blood. This result is also consistent with reports that have shown that JAM-C-deficient mice were more likely to have increased numbers of circulating neutrophils compared with their wild-type littermates^37^,^40^. The phenomenon may result from a defect in leukocyte migration^40^ and a low level of CXCR4 surface expression on granulocytes after an inflammatory challenge^37^ in these mice.

Cytokines play a major role in the pathogenesis of respiratory complications that follow AP^6^. The neutralization and blockade of cytokines reduces the severity of AP and attenuates the systemic stress response in this disorder^9^,^41^,^42^,^43^. In our study, the loss of JAM-C also resulted in significantly higher levels of serum TNF-α and IL-6 in experimental pancreatitis. TNF-α and IL-6 are secreted by neutrophils, monocytes, macrophages and lymphocytes^44^,^45^. Leukocytosis in JAM-C-deficient mice may contribute to the high levels of inflammatory cytokines in the circulation. Together, these results indicate that in SAP, the downregulation of JAM-C results in a severe systemic inflammatory response that may lead to ALI.

We have reported the occurrence of rTEM neutrophils under the conditions of reduced expression of JAM-C at endothelial cell junctions in a model of severe experimental pancreatitis. These neutrophils may contribute to ALI during AP and thus may represent a novel mechanism of AP-associated ALI. Notably, neutrophils that have undergone rTEM are also found in the blood of patients with AP. JAM-C may be considered a potential target for clinical applications, and thus, it would be useful to explore the role of these neutrophils in human AP further in future studies.

## Methods

### Mice

JAM-C-deficient (JAM-C^−/−^) mice on a C57BL/6 background were purchased from the Jackson Laboratory. The generation of JAM-C^−/−^ mice has been previously described^46^. Male knockout and wild-type mice that weighed 25-30 g at 8-10 weeks of age were used to investigate experimental pancreatitis. All Wild-type and JAM-C^−/−^ mice used in the study were weight matched. All animal experiments were approved by and were performed in accordance with the guidelines of the Animal Care and Use Committee of Shanghai Tongji University.

### Induction of experimental pancreatitis

Two AP models were applied in this study. Caerulein-pancreatitis was induced as previously described^26^. Briefly, mice were given ten hourly intraperitoneal injections of a supramaximal dose of caerulein (Sigma-Aldrich,St. Louis, Missouri, USA, 100 μg/kg). Lipopolysaccharide (LPS, Sigma-Aldrich, St. Louis, Missouri, USA, 5 mg/kg) was administered by intraperitoneal injection immediately after the 10th injection of caerulein. Mice were sacrificed 6h, 12h and 18h after the first caerulein injection. Arginine-pancreatitis was induced as previously reported^47^. Mice were intraperitoneally injected with L-arginine solution (Sigma-Aldrich, St. Louis, Missouri, USA, 8%, pH = 7.0) with an interval of 1h at a dose of 4 g/kg. Mice were sacrificed 3 days after the induction of acute pancreatitis. Peripheral blood, pulmonary vascular washout fluid, pancreatic tissue and lung tissue were collected.

### Leukocyte counts

Peripheral blood leukocyte counts were obtained for blood samples collected after retro-orbital puncture using heparinised capillary tubes. Blood samples were diluted 1:1 with PBS and 1.25 mM EDTA and counted using a Sysmex xe2100 haematology counter (Digitana, AG) as previously described^37^.

### Measurements of serum amylase and lipase

The serum activities of amylase and lipase were measured by enzyme dynamics chemistry using commercial kits according to the manufacturer’s protocols in a Roche/Hitachi modular analytics system (Roche, Mannheim, Germany).

### Measurement of serum TNF-α and IL-6

The levels of TNF-α and IL-6 in murine serum were analyzed using a commercially available ELISA kit (eBioscience, San Diego, CA, USA) according to the manufacturer’s instructions.

### Measurement of Myeloperoxidase Activity

Neutrophil sequestration in the lung tissue was quantified by measuring tissue MPO activity as previously reported^47^.

### Histological examination of the pancreas and lung

Tissues from the pancreas and lungs were fixed in 4% phosphate-buffered formaldehyde and stained with haematoxylin and eosin as we previously reported^48^. The samples were examined using light microscopy by two experienced observer who were blinded to the sample identities. The pancreas sections were scored for necrosis, oedema, and inflammation^48^ and the lung sections were scored for alveolar thickening and inflammation^49^ on a scale of 0 to 3 (0: the least severe and 3: the most severe) for each parameter.

### Immunohistochemistry

Paraformaldehyde-fixed, paraffin-embedded samples were sectioned at a thickness of 5 μm. Rat anti-mouse Ly-6G antibody (BioLegend) was used for immunohistochemistry according to established procedures^50^. The areas in all specimens that stained positive for Ly-6G were examined using a microscope (CTR 6000; Leica, Wetzlar, Germany).

### Flow cytometry

Peripheral blood was obtained from patients with AP, healthy volunteers and mice. Murine pulmonary washout was obtained as previously described^25^. Samples from patients were stained with antibodies against CD66b, CD16, ICAM-1 and CXCR1 or the respective isotype control antibodies. Murine samples were stained with antibodies against Ly-6G, ICAM-1 and CXCR1 or the respective isotype control antibodies. After incubation, erythrocytes were lysed in a lysis buffer (BD Biosciences), and the surface expression of molecules of interest was measured by flow cytometry, leukocytes were identified by forward and side light scatter characteristics. Neutrophils were identified in mice based on Ly-6G positivity and in humans based on positivity for both CD16 and CD66b. For each sample, 1×10^4^ neutrophils were analyzed for the expression of ICAM-1 and CXCR1. The following antibodies were used for flow cytometry: PE-conjugated rat anti-mouse Ly6G (BD Biosciences), FITC-conjugated hamster anti-mouse ICAM-1 (BD Biosciences), mouse monoclonal anti-CXCR1 (Abcam), PE/Cy7-conjugated goat anti-mouse IgG2a (Abcam), PE-conjugated mouse anti-human CD16 (BD Biosciences), PerCP-Cy5.5-conjugated mouse anti-human CD66b (BD Biosciences), APC-conjugated mouse anti-human ICAM-1 (BD Biosciences), FITC-conjugated mouse anti-human CXCR1 (BD Biosciences) and the respective isotype control antibodies (BD Biosciences).

### Immunofluorescence

Cryostat sections that were 5 μm thick were fixed in methanol and air-dried. A rabbit anti-mouse PECAM-1 (Abcam) and goat anti-mouse JAM-C (R&D Systems) antibodies were used for immunofluorescence as previously reported^26^. Pictures were acquired using a Zeiss LSM510 laser scanning confocal microscope. Quantitative analysis of staining for JAM-C was performed by Image Pro Plus 6.0.

### Western blotting

Frozen pancreatic tissue samples were ground to a powder in liquid nitrogen and then reconstituted in ice-cold RIPA buffer with 1 mmol/L phenylmethanesulphonyl fluoride and protease inhibitors (Sigma-Aldrich). The tissue homogenates were centrifuged and the supernatants collected. Samples (80 μg per lane) were analyzed by gel electrophoresis, using a 10% acrylamide gel in Tris–HCl buffer, and were then transferred electrophoretically to PVDF membranes. Murine JAM-C was detected using a goat anti-mouse JAM-C antibody (R&D System). Labelled proteins were visualised with an HRP-coupled secondary antibody. The optical densities of the protein lanes were measured using an Odyssey scanning system, and the JAM-C values were adjusted relative to the levels of actin expression.

### Real-time quantitative reverse transcription PCR

Total RNA was extracted from each tissue sample using the acid guanidinium/phenol/chloroform method as previously described^48^. RNA was reverse transcribed and subjected to real-time PCR using gene-specific, intron-spanning primers (Table 5). qRT-PCR was performed in triplicate for each gene of interest under each experimental condition using an ABI Prism 7900HT Sequence Detection System (Applied Biosystems, CA, USA). Fold changes and gene expression levels were calculated using the comparative CT (2^−ΔΔCT^) method.

### Patients with AP

Of the patients who were admitted to the Gastroenterology Department of the Shanghai Tenth People’s Hospital from December 2013 to June 2014, those who met the diagnostic criteria of AP^51^ were included in the study. Patients with chronic or other acute inflammatory diseases were excluded. Healthy volunteers were assessed as the control group. The severity of AP was assessed on the basis of the revised Atlanta criteria^51^. Arterial oxygen pressures and the inspired oxygen concentration ratio (PaO2/FiO2) were recorded to evaluate the extent of lung injury; a value below 300 defines the presence of ALI. The entire study design and procedures involved were in accordance with the Declaration of Helsinki. Informed written consent was obtained from all AP patients and healthy volunteers. The study protocol was approved by the medical ethics committee of Shanghai Tenth People’s Hospital. The methods regarding human subjects were carried out in accordance with approved guidelines and regulations.

### Data presentation and statistics

The data are presented as the mean ± SEM. Comparisons between the group means were performed using the Mann-Whitney non-parametric U test. Correlation analyses were performed by linear regression. Values of *p* < 0.05 were considered statistically significant.

## Additional Information

**How to cite this article**: Wu, D. *et al.* Reverse-migrated neutrophils regulated by JAM-C are involved in acute pancreatitis-associated lung injury. *Sci. Rep.* **6**, 20545; doi: 10.1038/srep20545 (2016).

## Supplementary Material

Supplementary Information

## Figures and Tables

**Table 1:** Histological evaluation of pancreatic and lung injuries between caerulein plus LPS and single caerulein-induced pancreatitis.

	NC (n = 6)	caerulein (n = 6)	caerulein+LPS (n = 6)
pancreas			
Oedema	0 ± 0	2.34 ± 0.15	2.39 ± 0.23
Inflammatory cell infiltrate	0 ± 0	1.80 ± 0.11	2.22 ± 0.31*
Necrosis	0 ± 0	1.60 ± 0.19	1.94 ± 0.23*
lung			
Alveolar thickening	0 ± 0	1.23 ± 0.33	1.78 ± 0.23*
Inflammation	0 ± 0	1.27 ± 0.35	1.72 ± 0.29*

^*^p < 0.05 versus caerulein group.

**Table 2:** Histological evaluation of lung injuries following caerulein and LPS-induced pancreatitis.

Time (h)	0 h (n = 6)	6 h (n = 6)	12 h (n = 6)	18 h (n = 6)
Alveolar thickening	0 ± 0	0.67 ± 0.23	1.78 ± 0.23*	1.83 ± 0.24*
Inflammation	0 ± 0	0.58 ± 0.23	1.72 ± 0.29*	1.80 ± 0.32*

^*^p < 0.05 versus 6 h group.

**Table 3:** Histological evaluation of pancreatic damage in JAM-C knockout mice following experimental acute pancreatitis.

	caerulein+LPS			L-Arg		
	NC (n = 4)	Wild-type (n = 6)	JAM-C^−/−^ (n = 11)	NC (n = 4)	Wild-type (n = 7)	JAM-C^−/−^ (n = 7)
Oedema	0 ± 0	2.39 ± 0.23	2.41 ± 0.24*	0 ± 0	2.57 ± 0.29	2.54 ± 0.15*
Inflammatory cell infiltrate	0 ± 0	2.22 ± 0.31	2.24 ± 0.30*	0 ± 0	2.63 ± 0.37	2.60 ± 0.15*
Necrosis	0 ± 0	1.94 ± 0.23	1.86 ± 0.25*	0 ± 0	2.76 ± 0.38	2.50 ± 0.22*

NC: normal control, *p > 0.05 versus wild-type mice in the same AP model.

**Table 4:** Histological evaluation of lung injuries in JAM-C knockout mice following experimental acute pancreatitis.

	caerulein+LPS			L-Arg		
	NC (n = 4)	Wild-type (n = 6)	JAM-C^−/−^ (n = 11)	NC (n = 4)	Wild-type (n = 7)	JAM-C^−/−^(n = 7)
Alveolar thickening	0 ± 0	1.78 ± 0.23	2.65 ± 0.12*	0 ± 0	2.03 ± 0.22	2.71 ± 0.12*
Inflammation	0 ± 0	1.72 ± 0.29	2.53 ± 0.22*	0 ± 0	1.97 ± 0.32	2.54 ± 0.25*

NC: normal control, *p < 0.05 versus wild-type mice in the same AP model.

**Table 5:** Primer sequences used for qRT-PCR analysis.

Genes	Primers	Size (bp)	Annealing temperature (°C)
TNF-α	F: 5′-GGGTGTATGGCCTCATCAC-3′ R: 5′-ACAGGCAGTGATCAGGAACT-3′	260	51
IL-6	F: 5′-CCACTGCCTTCCCTACTT-3′ R: 5′-TTGCCATTGCACAACTCTT-3′	154	50
GAPDH	F: 5′-GCAAGTTCAACGGCACAG-3′ R: 5′-CGCCAGTAGACTCCACGAC-3′	141	52

## References

[b1] Agarwal N. & Pitchumoni C. S. Acute pancreatitis: a multisystem disease. Gastroenterologist. 1, 115–128 (1993). 8049885

[b2] Johnson C. D. & Abu-Hilal M. Persistent organ failure during the first week as a marker of fatal outcome in acute pancreatitis. Gut 53, 1340–1344 (2004). 15306596 10.1136/gut.2004.039883 PMC1774183

[b3] Mofidi R. *et al.* Association between early systemic inflammatory response, severity of multiorgan dysfunction and death in acute pancreatitis. Br. J. Surg. 93, 738–744 (2006). 16671062 10.1002/bjs.5290

[b4] Buter A., Imrie C. W., Carter C. R., Evans S. & McKay C. J. Dynamic nature of early organ dysfunction determines outcome in acute pancreatitis. Br. J. Surg. 89, 298–302 (2002). 11872053 10.1046/j.0007-1323.2001.02025.x

[b5] Andersson B., Ansari D., Andersson E., Persson S. & Andersson R. Fatal acute pancreatitis occurring outside of the hospital: clinical and social characteristics. World. J. Surg. 34, 2286–2291 (2010). 20574639 10.1007/s00268-010-0693-z

[b6] Pastor C. M. & Frossard J. L. Are genetically modified mice useful for the understanding of acute pancreatitis? FASEB. J. 15, 893–897 (2001). 11292648 10.1096/fj.00-0672rev

[b7] Denham W. *et al.* Inhibition of p38 mitogen activate kinase attenuates the severity of pancreatitis-induced adult respiratory distress syndrome. Crit. Care. Med. 28, 2567–2572 (2000). 10921596 10.1097/00003246-200007000-00064

[b8] Denham W., Yang J. & Norman J. Evidence for an unknown component of pancreatic ascites that induces adult respiratory distress syndrome through an interleukin-1 and tumor necrosis factor-dependent mechanism. Surgery 122, 295–301, discussion 301–292 (1997). 9288135 10.1016/s0039-6060(97)90021-0

[b9] Bhatia M. *et al.* Inflammatory mediators in acute pancreatitis. J. Pathol. 190, 117–125 (2000). 10657008 10.1002/(SICI)1096-9896(200002)190:2<117::AID-PATH494>3.0.CO;2-K

[b10] Grewal H. P. *et al.* Induction of tumor necrosis factor in severe acute pancreatitis and its subsequent reduction after hepatic passage. Surgery 115, 213–221 (1994). 8310410

[b11] Tsukahara Y. *et al.* Role of nitric oxide derived from alveolar macrophages in the early phase of acute pancreatitis. J. Surg. Res. 66, 43–50 (1996). 8954830 10.1006/jsre.1996.0370

[b12] Closa D. *et al.* Activation of alveolar macrophages in lung injury associated with experimental acute pancreatitis is mediated by the liver. Ann. Surg. 229, 230–236 (1999). 10024105 10.1097/00000658-199902000-00011 PMC1191636

[b13] Dugernier T., Laterre P. F. & Reynaert M. S. Ascites fluid in severe acute pancreatitis: from pathophysiology to therapy. Acta. Gastroenterol. Belg. 63, 264–268 (2000). 11189983

[b14] Gloor B. *et al.* Kupffer cell blockade reduces hepatic and systemic cytokine levels and lung injury in hemorrhagic pancreatitis in rats. Pancreas 21, 414–420 (2000). 11075997 10.1097/00006676-200011000-00013

[b15] Gea-Sorli S. & Closa D. Role of macrophages in the progression of acute pancreatitis. World. J. Gastrointest. Pharmacol. Ther. 1, 107–111 (2010). 21577304 10.4292/wjgpt.v1.i5.107 PMC3091151

[b16] Yang J., Denham W., Carter G., Tracey K. J. & Norman J. Macrophage pacification reduces rodent pancreatitis-induced hepatocellular injury through down-regulation of hepatic tumor necrosis factor alpha and interleukin-1beta. Hepatology 28, 1282–1288 (1998). 9794913 10.1002/hep.510280517

[b17] Yang J. *et al.* The physiologic consequences of macrophage pacification during severe acute pancreatitis. Shock 10, 169–175 (1998). 9744644 10.1097/00024382-199809000-00004

[b18] Tahamont M. V., Barie P. S., Blumenstock F. A., Hussain M. H. & Malik A. B. Increased lung vascular permeability after pancreatitis and trypsin infusion. Am. J. Pathol. 109, 15–26 (1982). 6181692 PMC1916070

[b19] Lungarella G., Gardi C., de Santi M. M. & Luzi P. Pulmonary vascular injury in pancreatitis: evidence for a major role played by pancreatic elastase. Exp. Mol. Pathol. 42, 44–59 (1985). 3843961 10.1016/0014-4800(85)90017-6

[b20] Feddersen C. O. *et al.* Lung injury in acute experimental pancreatitis in rats. II. Functional studies. Int. J. Pancreatol. 8, 323–331 (1991). 1791318 10.1007/BF02952724

[b21] Willemer S., Feddersen C. O., Karges W. & Adler G. Lung injury in acute experimental pancreatitis in rats. I. Morphological studies. Int. J. Pancreatol. 8, 305–321 (1991). 1791317 10.1007/BF02952723

[b22] Guice K. S., Oldham K. T., Caty M. G., Johnson K. J. & Ward P. A. Neutrophil-dependent, oxygen-radical mediated lung injury associated with acute pancreatitis. Ann. Surg. 210, 740–747 (1989). 2589887 10.1097/00000658-198912000-00008 PMC1357865

[b23] Bhatia M. *et al.* The effects of neutrophil depletion on a completely noninvasive model of acute pancreatitis-associated lung injury. Int. J. Pancreatol. 24, 77–83 (1998). 9816540 10.1007/BF02788564

[b24] Buckley C. D. *et al.* Identification of a phenotypically and functionally distinct population of long-lived neutrophils in a model of reverse endothelial migration. J. Leukoc. Biol. 79, 303–311 (2006). 16330528 10.1189/jlb.0905496

[b25] Woodfin A. *et al.* The junctional adhesion molecule JAM-C regulates polarized transendothelial migration of neutrophils *in vivo*. Nat. Immunol. 12, 761–769 (2011). 21706006 10.1038/ni.2062 PMC3145149

[b26] Vonlaufen A. *et al.* The role of junctional adhesion molecule C (JAM-C) in acute pancreatitis. J. Pathol. 209, 540–548 (2006). 16767690 10.1002/path.2007

[b27] Power C., Wang J. H., Sookhai S., Wu Q. D. & Redmond H. P. Proinflammatory effects of bacterial lipoprotein on human neutrophil activation status, function and cytotoxic potential *in vitro*. Shock 15, 461–466 (2001). 11386619 10.1097/00024382-200115060-00009

[b28] Wang J. H. *et al.* Intercellular adhesion molecule-1 (ICAM-1) is expressed on human neutrophils and is essential for neutrophil adherence and aggregation. Shock 8, 357–361 (1997). 9361346 10.1097/00024382-199711000-00007

[b29] Jacobs M. L. *et al.* Acute pancreatitis: analysis of factors influencing survival. Ann. Surg. 185, 43–51 (1977). 831635 10.1097/00000658-197701000-00007 PMC1396261

[b30] Pastor C. M., Matthay M. A. & Frossard J. L. Pancreatitis-associated acute lung injury: new insights. Chest 124, 2341–2351 (2003). 14665518 10.1378/chest.124.6.2341

[b31] Shields C. J., Winter D. C. & Redmond H. P. Lung injury in acute pancreatitis: mechanisms, prevention, and therapy. Curr. Opin. Crit. Care. 8, 158–163 (2002). 12386518 10.1097/00075198-200204000-00012

[b32] Zhao X., Andersson R., Wang X., Dib M. & Wang X. Acute pancreatitis-associated lung injury: pathophysiological mechanisms and potential future therapies. Scand. J. Gastroenterol. 37, 1351–1358 (2002). 12523582 10.1080/003655202762671206

[b33] Bradfield P. F. *et al.* JAM-C regulates unidirectional monocyte transendothelial migration in inflammation. Blood 110, 2545–2555 (2007). 17625065 10.1182/blood-2007-03-078733 PMC1988941

[b34] Chavakis T. *et al.* The junctional adhesion molecule-C promotes neutrophil transendothelial migration *in vitro* and *in vivo*. J. Biol. Chem. 279, 55602–55608 (2004). 15485832 10.1074/jbc.M404676200

[b35] Aurrand-Lions M. *et al.* Junctional adhesion molecule-C regulates the early influx of leukocytes into tissues during inflammation. J. Immunol. 174, 6406–6415 (2005). 15879142 10.4049/jimmunol.174.10.6406

[b36] Ludwig R. J. *et al.* Junctional adhesion molecules (JAM)-B and -C contribute to leukocyte extravasation to the skin and mediate cutaneous inflammation. J. Invest. Dermatol. 125, 969–976 (2005). 16297198 10.1111/j.0022-202X.2005.23912.x

[b37] Imhof B. A. *et al.* Pulmonary dysfunction and impaired granulocyte homeostasis result in poor survival of Jam-C-deficient mice. J. Pathol. 212, 198–208 (2007). 17455169 10.1002/path.2163

[b38] Mandicourt G., Iden S., Ebnet K., Aurrand-Lions M. & Imhof B. A. JAM-C regulates tight junctions and integrin-mediated cell adhesion and migration. J. Bio. Chem. 282, 1830–1837 (2007). 17099249 10.1074/jbc.M605666200

[b39] Orlova V. V., Economopoulou M., Lupu F., Santoso S. & Chavakis T. Junctional adhesion molecule-C regulates vascular endothelial permeability by modulating VE-cadherin-mediated cell-cell contacts. J. Exp. Med. 203, 2703–2714 (2006). 17116731 10.1084/jem.20051730 PMC2118160

[b40] Praetor A. *et al.* Genetic deletion of JAM-C reveals a role in myeloid progenitor generation. Blood 113, 1919–1928 (2009). 19109565 10.1182/blood-2008-06-159574 PMC2651011

[b41] Fink G., Yang J., Carter G. & Norman J. Acute pancreatitis-induced enzyme release and necrosis are attenuated by IL-1 antagonism through an indirect mechanism. J. Surg. Res. 67, 94–97 (1997). 9070189 10.1006/jsre.1996.4935

[b42] Norman J. G. *et al.* Tissue-specific cytokine production during experimental acute pancreatitis. A probable mechanism for distant organ dysfunction. Dig. Dis. Sci. 42, 1783–1788 (1997). 9286248 10.1023/a:1018886120711

[b43] Kaplan M. *et al.* Effectiveness of interleukin-1 receptor antagonist (Anakinra) on cerulein-induced experimental acute pancreatitis in rats. Scand. J. Gastroenterol. 49, 1124–1130 (2014). 24912987 10.3109/00365521.2014.926983

[b44] Witko-Sarsat V., Rieu P., Descamps-Latscha B., Lesavre P. & Halbwachs-Mecarelli L. Neutrophils: molecules, functions and pathophysiological aspects. Lab. Invest. 80, 617–653 (2000). 10830774 10.1038/labinvest.3780067

[b45] Olszewski M. B., Groot A. J., Dastych J. & Knol E. F. TNF trafficking to human mast cell granules: mature chain-dependent endocytosis. J. Immunol. 178, 5701–5709 (2007). 17442953 10.4049/jimmunol.178.9.5701

[b46] Gliki G., Ebnet K., Aurrand-Lions M., Imhof B. A. & Adams R. H. Spermatid differentiation requires the assembly of a cell polarity complex downstream of junctional adhesion molecule-C. Nature 431, 320–324 (2004). 15372036 10.1038/nature02877

[b47] Dawra R. *et al.* Development of a new mouse model of acute pancreatitis induced by administration of L-arginine. Am. J. Physiol. Gastrointest. Liver Physiol. 292, G1009–1018 (2007). 17170029 10.1152/ajpgi.00167.2006

[b48] Hu G. *et al.* Reg4 protects against acinar cell necrosis in experimental pancreatitis. Gut 60, 820–828 (2011). 21193457 10.1136/gut.2010.215178

[b49] Bhatia M. *et al.* Treatment with neutralising antibody against cytokine induced neutrophil chemoattractant (CINC) protects rats against acute pancreatitis associated lung injury. Gut 47, 838–844 (2000). 11076884 10.1136/gut.47.6.838 PMC1728153

[b50] Lamagna C., Hodivala-Dilke K. M., Imhof B. A. & Aurrand-Lions M. Antibody against junctional adhesion molecule-C inhibits angiogenesis and tumor growth. Cancer. Res. 65, 5703–5710 (2005). 15994945 10.1158/0008-5472.CAN-04-4012

[b51] Banks P. A. *et al.* Classification of acute pancreatitis–2012: revision of the Atlanta classification and definitions by international consensus. Gut 62, 102–111 (2013). 23100216 10.1136/gutjnl-2012-302779

