# Supplementary material for: Reverse-migrated neutrophils regulated by JAM-C are involved in acute pancreatitis-associated lung injury
Source: Sci Rep. 2016 Feb 4;6:20545. doi: 10.1038/srep20545 (PMC4740794; doi:10.1038/srep20545)
Supplement: Supplementary Information [file srep20545-s1.doc]

**Reverse-migrated neutrophils regulated by JAM-C are involved in acute pancreatitis-associated lung injury**

Deqing Wu^1^, Yue Zeng^2^, Yuting Fan^1^, Jianghong Wu ^2^, Tunike Mulatibieke^2^, Jianbo Ni^2^, Ge Yu^2^, Rong Wan^2^, Wang Xingpeng^1, 2^*****,Guoyong Hu^2^*****

1. Department of Gastroenterology, Shanghai Tenth People’s Hospital, Tongji University School of Medicine, Shanghai, China

2. Department of Gastroenterology, Shanghai General Hospital, Shanghai Jiaotong University School of Medicine, Shanghai, China

*****Correspondence to

Xingpeng Wang, Department of Gastroenterology, Shanghai Tenth People’s Hospital, Tongjii University School of Medicine, 301 Yanchang Road, Zhabei District, Shanghai, China. Department of Gastroenterology, Shanghai General Hospital, Shanghai Jiaotong University School of Medicine, 100 Haining Road, Hongkou District, Shanghai, China. E-mail: richardwangxp@163.com

Guoyong Hu, Department of Gastroenterology, Shanghai General Hospital, Shanghai Jiaotong University School of Medicine, 100 Haining Road, Hongkou District, Shanghai, China. E-mail: [huguoyongsh@sina.com](mailto:huguoyongsh@sina.com)

**Supporting Online Material**

Supplementary Figure and legend

**Supplementary Figure S1**

**Expression of JAM-C in pancreas by immunofluorescence during caerulein and LPS-induced pancreatitis.**


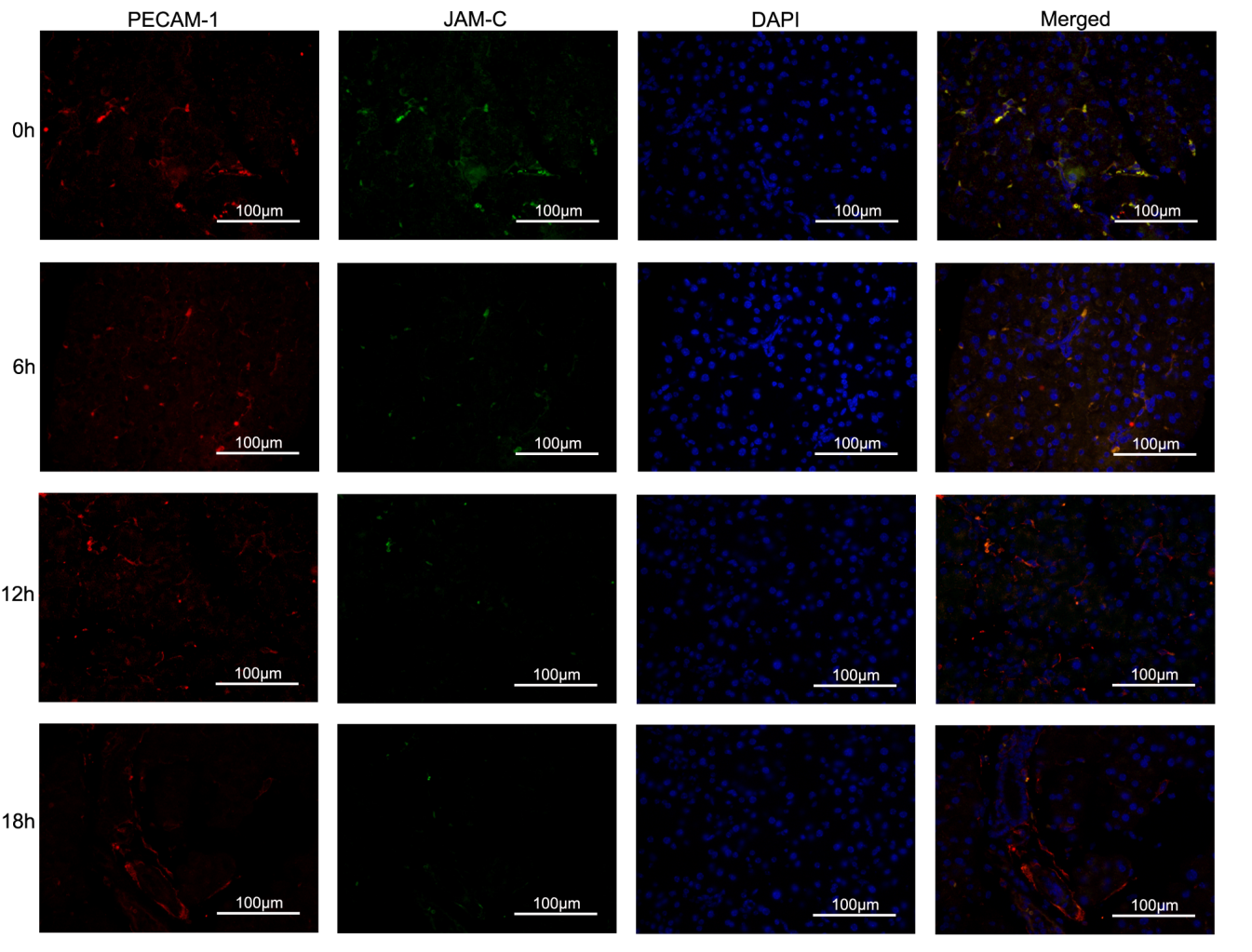
JAM-C protein expression (green) is shown on immunofluorescence with a co-staining for PECAM-1 (red) by representative micrographs. DAPI (blue) was used to counterstain the nuclei. JAM-C is expressed in blood vessels in the murine pancreas. The level of JAM-C expression is downregulated at both 12 h and 18 h after the first caerulein injection. Original magnification: ×400. Mice were sacrificed 0 h, 6 h, 12 h and 18 h after the first caerulein injection.
